# Birefringence-derived scleral artifacts in optical coherence tomography images of eyes with pathologic myopia

**DOI:** 10.1038/s41598-022-23874-7

**Published:** 2022-11-16

**Authors:** Masahiro Miura, Shuichi Makita, Yoshiaki Yasuno, Atsuya Miki, Rei Nemoto, Hiroyuki Shimizu, Shinnosuke Azuma, Toshihiro Mino, Tatsuo Yamaguchi

**Affiliations:** 1 Department of Ophthalmology, Ibaraki Medical Center, Tokyo Medical University, 3-20-1 Chuo, Ami, Inashiki, Ibaraki 300395 Japan; 2 Computational Optics Group, University of Tsukuba, Tsukuba, Japan; 3 Department of Myopia Control Research, Aichi Medical University, Nagakute, Japan; 4 Topcon Corporation, Tokyo, Japan

**Keywords:** Medical research, Translational research, Eye diseases, Retinal diseases

## Abstract

We investigated birefringence-derived scleral artifacts in optical coherence tomography (OCT) images of eyes with pathologic myopia. This study included 76 eyes of 42 patients with pathologic myopia. Five sets of OCT B-scan images of the macula were obtained using commercial swept-source OCT. A dataset of prototype swept-source polarization-diversity OCT images was used to identify polarization-dependent OCT images (i.e., complex averaging of OCT signals from two polarization channels) and polarization-independent OCT images (i.e., intensity averaging of two OCT signals). Polarization-dependent OCT images and commercial OCT images were assessed for the presence of birefringence-derived artifacts by comparison with polarization-independent OCT images. Both polarization-dependent OCT images and commercial OCT images contained scleral vessel artifacts. Scleral vessel artifacts were present in 46 of 76 eyes (60.5%) imaged by polarization-dependent OCT and 17 of 76 eyes (22.4%) imaged by commercial OCT. The proportion of images that showed scleral vessel artifacts was significantly greater among polarization-dependent OCT images than among commercial OCT images (*P* < 0.001). Additionally, polarization-dependent OCT images showed low-intensity band artifacts. This study demonstrated the existence of birefringence-derived scleral artifacts in commercial OCT images and indicated that polarization-diversity OCT is an effective tool to evaluate the presence of these artifacts.

## Introduction

The sclera is a fibrous connective tissue that consists of anisotropically arranged collagen lamellae^1,2^. The sclera provides a strong framework to protect the contents of the eye from external trauma and intraocular pressure-induced distension^2^. Previous studies have shown that the sclera is involved in the development of ocular diseases such as pathologic myopia and glaucoma^2–4^. Therefore, clinical evaluations of scleral structure are necessary to identify its relationships with visual function.

Recent advances in optical coherence tomography (OCT) technology have enabled clinical evaluations of the human sclera in eyes with pathologic myopia^5–12^. Observations of posterior scleral structure are generally easier in eyes with pathologic myopia than in healthy eyes because signal attenuation is reduced as choroidal thickness decreases^6^. Clinical evaluations of scleral thickness and scleral vessels in eyes with pathologic myopia have been performed using swept-source OCT with a 1-µm wavelength band^5,6,9–12^ or enhanced depth imaging involving spectral-domain OCT with an 850-nm wavelength band^7,8^. These studies provided important information regarding pathologic myopia. Notably, possible associations of scleral thickness with pathologic myopia have been reported^9,12^. Evaluations of scleral vessels were performed to identify ciliary artery variations in eyes with pathologic myopia^5,7,8,11^ and explore the development of myopic choroidal neovascularization^10^.

The sclera exhibits local birefringence because of dense collagen lamellae^13–15^. This birefringence alters the polarization state of the incident light. In conventional OCT, the intensity of the OCT signal is altered because of the relative difference in polarization states between the incident light and the reference light during interferometric measurements. This alteration of OCT signals can cause artifacts (i.e., artifactual structures) in OCT images. The polarization-diversity detection technique, which is frequently used in polarization-sensitive OCT^16,17^, can remove these birefringence-derived artifacts^18–22^. A previous polarization-sensitive OCT study revealed artifactual linear structures that appeared to be scleral vessels^18^. The presence of low-intensity band artifacts in OCT images of the sclera has also been reported^19,20^. To our knowledge, available retinal commercial OCT systems are polarization-dependent; thus, birefringence-derived artifacts might exist in commercial OCT images of the sclera^20^. These artifactual structures can easily be distinguished from real scleral structures using datasets of polarization-diversity OCT (PD-OCT) images^18–20^.

The purposes of this study were to evaluate artifacts derived from scleral birefringence in eyes with pathologic myopia using PD-OCT and commercial OCT and to investigate the clinical significance of these scleral artifacts in OCT images.

## Methods

This prospective, observational, cross-sectional study was performed using a protocol that adhered to the tenets of the Declaration of Helsinki. The study was approved by the Institutional Review Board of Tokyo Medical University (approval number: T2019-0072). The study was registered in the University Hospital Medical Information Network database (UMIN 000,039,650; http://www.umin.ac.jp/ctr/index-j.htm). The nature of the study and the implications of participation were explained to all potential participants. Written informed consent was obtained from each participant before any study procedures or examinations were performed.

We examined 76 eyes of 42 patients (10 men, 32 women; age range, 23–82 years; mean age, 62.0 years) (Supplementary Table S1) with pathologic myopia (axial length of ≥ 26.5 mm). Axial length was measured using an optical biometer (OA-2000; Tomey, Nagoya, Japan); the mean axial length was 29.0 mm (range, 26.5–32.4 mm). Lesions associated with pathologic myopia in this study were active myopic choroidal neovascularization (10 eyes), myopic choroidal neovascularization in remission (24 eyes), macular retinoschisis with myopic choroidal neovascularization in remission (1 eye), a history of macular hole surgery (1 eye), and macular hole before treatment (1 eye).

**Figure 1:**
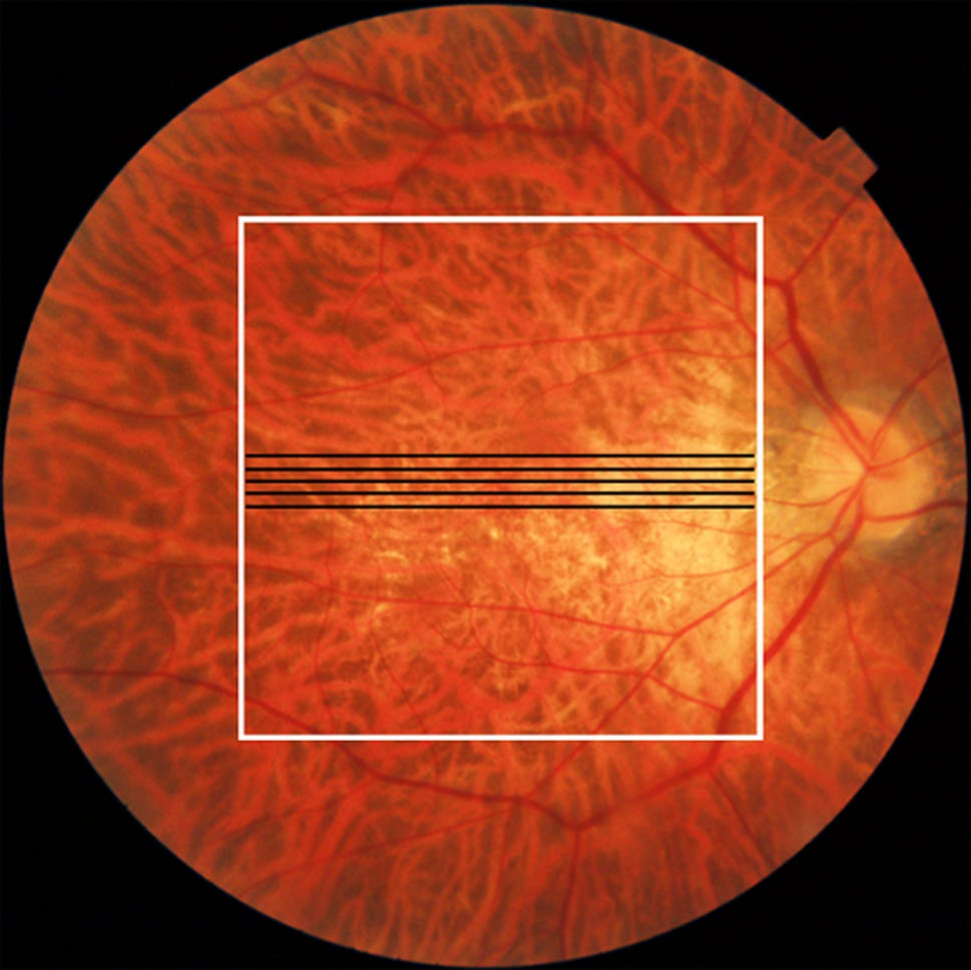
Measurement area in a color fundus image of the right eye of a 50-year-old woman with pathologic myopia. Black lines indicate five sets of macular scan lines of commercial OCT. The white line indicates the measurement area of PD-OCT.

Commercial OCT measurement was performed using a DRI-OCT Triton system (Topcon Corp., Tokyo, Japan). This commercial OCT system is a polarization-dependent swept-source OCT device with a 1-μm wavelength band. In this context, polarization-dependent means that the device captures only one of the two polarization components of the probe beam; thus, it can be affected by birefringence-derived artifacts. The axial scan speed was 100,000 A-scans/s. The catalog-specified axial resolution was 8 μm in the tissue. Five sets of macular horizontal B-scans in five-line cross mode were used to collect images of the sclera (Fig. 1). The length of each B-scan was 6 mm, and the vertical interval between each set of B-scan images was 0.15 mm. Each B-scan image consisted of 1024 A-scans, and the depth range for each B-scan image was 2.6 mm in the tissue. Each B-scan image was constructed by averaging 16 consecutive B-scan images.

A detailed description of the prototype PD-OCT hardware was previously published^23^. The PD-OCT system is a swept-source OCT device with a 1-μm wavelength band and polarization-diversity detection; it enables measurement of the polarization state of backscattered light from the eye. The axial scan speed was 100,000 A-scans/s, and the depth range of each B-scan was 2.8 mm in the tissue. The axial resolution was measured as 6 μm in the tissue. This PD-OCT system used an OCT retinal scanner that was modified from DRI-OCT Atlantis (Topcon Corp.).

From the dataset of PD-OCT images, two types of scattering OCT images were constructed. The first set of images was constructed by complex averaging of OCT signals from two detection channels of the polarization diverse detector, which corresponded to two orthogonal polarization components of the detected light. The complex averaging process mimics the following physical process. The probe beam is split into polarization channels, which are combined in the form of the complex field, and this combined field is identical to the original physical probe beam before splitting. Similarly, this combination process reconstructs the original reference beam. Because the complex averaged signal is the summation of these virtually reconstructed original probe and reference beams, it is identical to the standard OCT signal. Hence, the complex averaged OCT images exhibit birefringence-derived artifacts (polarization-dependent OCT images) similar to the artifacts present in single-detector OCT images (i.e., images from conventional commercial OCT).

The second set of images was constructed by intensity averaging of the two OCT signals [Eq. (5) of Ref.^23^]. When the reference beam powers of the two channels are properly balanced, the intensity of the intensity-averaging signal becomes proportional to the total power of the backscattered probe beam, regardless of the polarization state of the backscattered probe beam. The intensity-averaging signal is thus insensitive to the polarization state variation of the backscattered probe beam. Accordingly, the intensity-averaged OCT images are free of birefringence-derived artifacts (polarization-independent OCT images).

Volumetric scans were performed using a raster scanning protocol with 512 A-lines × 256 B-scans that covered a 6.0- × 6.0-mm region of the retina (Fig. 1). From a series of 256 B-scan images of PD-OCT images, PD-OCT images (polarization-dependent and -independent) were manually selected at locations that corresponded to five sets of commercial OCT images.

Birefringence-derived scleral artifacts in OCT images were then evaluated using PD-OCT images and commercial OCT images. First, real scleral structures free of birefringence-derived artifacts were evaluated using polarization-independent OCT images. Second, birefringence-derived artifacts in polarization-dependent OCT images and commercial OCT images were evaluated by comparison with polarization-independent OCT images. Artifactual structures were classified as scleral vessel artifacts or low-intensity band artifacts. Scleral vessel artifacts were defined as artifactual linear low-intensity structures that coursed obliquely within the sclera. Low-intensity band artifacts were defined as artifactual thick low-intensity bands that coursed parallel to the scleral curvature. Two ophthalmologists (M.M. and H.S.) subjectively evaluated real scleral structures in polarization-independent OCT images, as well as artifactual structures in polarization-dependent OCT images and commercial OCT images. Cases of disagreement concerning the subjective evaluations were resolved by consensus after discussion with a third ophthalmologist (R.N.). The reproducibility of all artifacts in commercial OCT was evaluated by comparison of two successively repeated measurements in the same measurement session.

To evaluate artifacts throughout the PD-OCT measurement area, a series of 256 PD-OCT B-scan images was thoroughly evaluated to identify such artifacts in polarization-dependent OCT images. Subsequently, *en face* distributions of artifactual structures were evaluated using an automatic segmentation method that was identical to the built-in software in a commercially available OCT device (DRI-OCT Triton). Each PD-OCT B-scan image was flattened with respect to Bruch’s membrane to generate a reference plane for *en face* display. *En face* displays of both polarization-independent OCT images and polarization-dependent OCT images were constructed in the same scleral plane to evaluate the *en face* distributions of artifactual structures.

Statistical analyses were performed with IBM SPSS Statistics for Windows, version 28.0 (IBM Corp., Armonk, NY, USA). Statistical significance was defined as *P* < 0.05.

## Results

**Figure 2:**
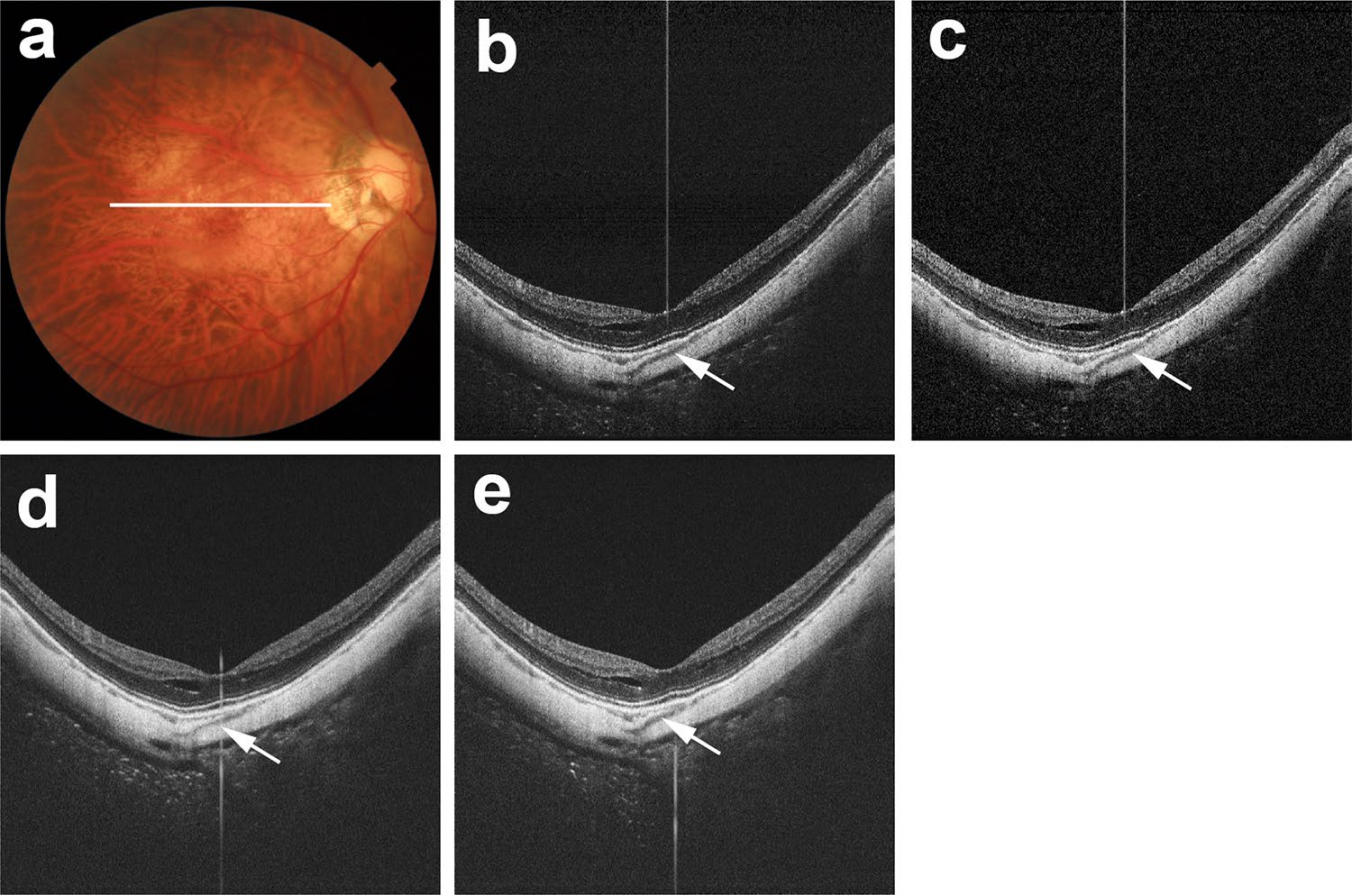
Real scleral vessels without artifactual structures in PD-OCT images and commercial OCT images. The right eye of a 56-year-old woman with pathologic myopia is shown. White line in color fundus image (**a**) indicates the scan line for PD-OCT images and commercial OCT B-scan images (**b**–**e**). (**b**) Polarization-independent OCT B-scan image (**b**) shows a real scleral vessel (white arrow). Polarization-dependent OCT B-scan image (**c**) and commercial OCT B-scan images from two repeated measurements (**d**, **e**) also show real scleral vessels (white arrows) without scleral vessel artifacts. The vertical lines in B-scan images (**b**–**e**) are artifacts by the reflection from a lens inside the OCT system coming into the images as coherent revival noise.

**Figure 3:**
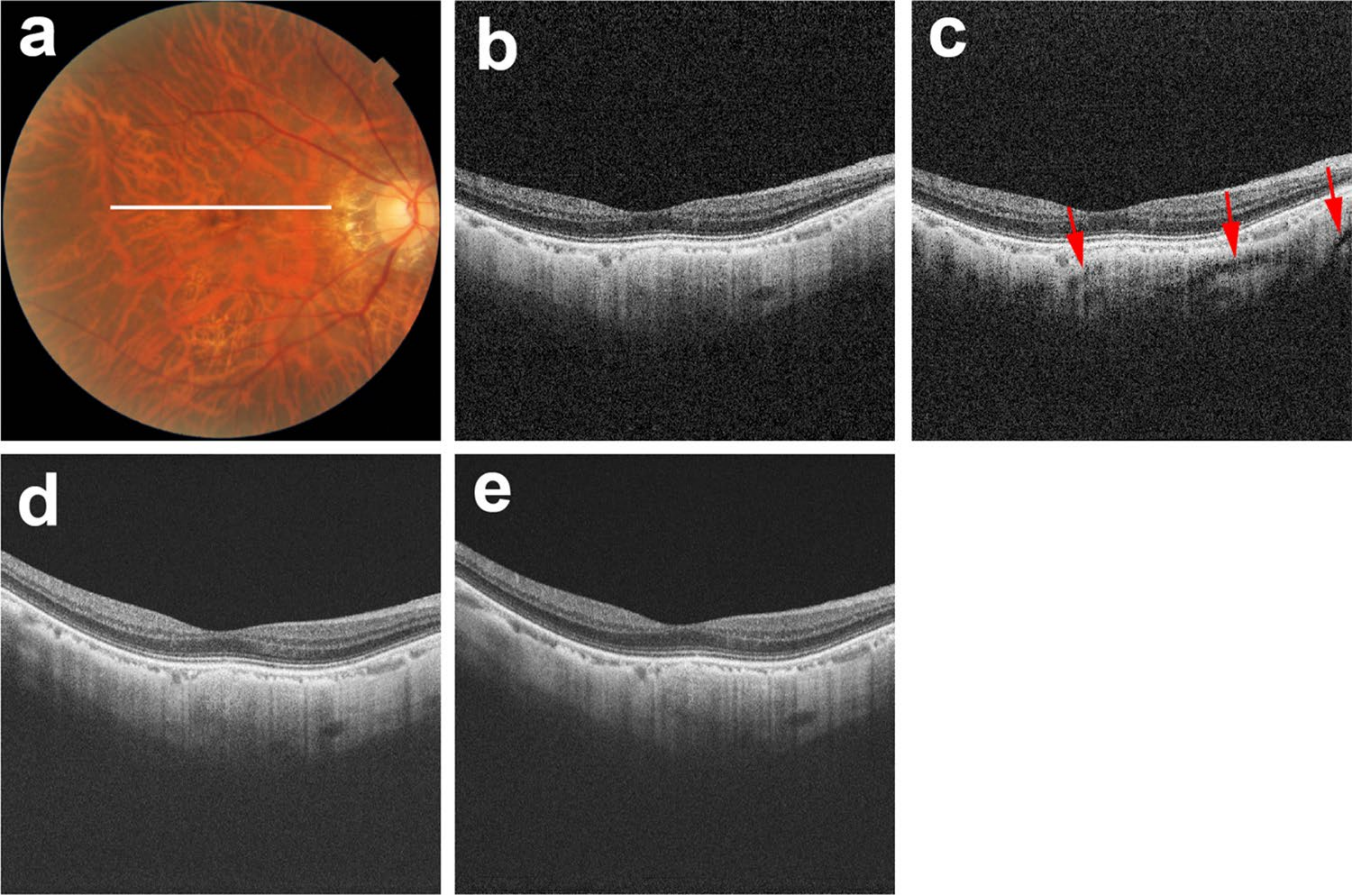
Scleral vessel artifacts in a polarization-dependent OCT image. The right eye of a 66-year-old woman with pathologic myopia is shown. White line in color fundus image (**a**) indicates the scan line for PD-OCT images and commercial OCT B-scan images (**b**–**e**). Polarization-dependent OCT B-scan image (**c**) shows scleral vessel artifacts (red arrows). No scleral vessel artifacts are present in a polarization-independent OCT B-scan image (**b**) or in two repeated commercial OCT B-scan images (**d**, **e**).

**Figure 4:**
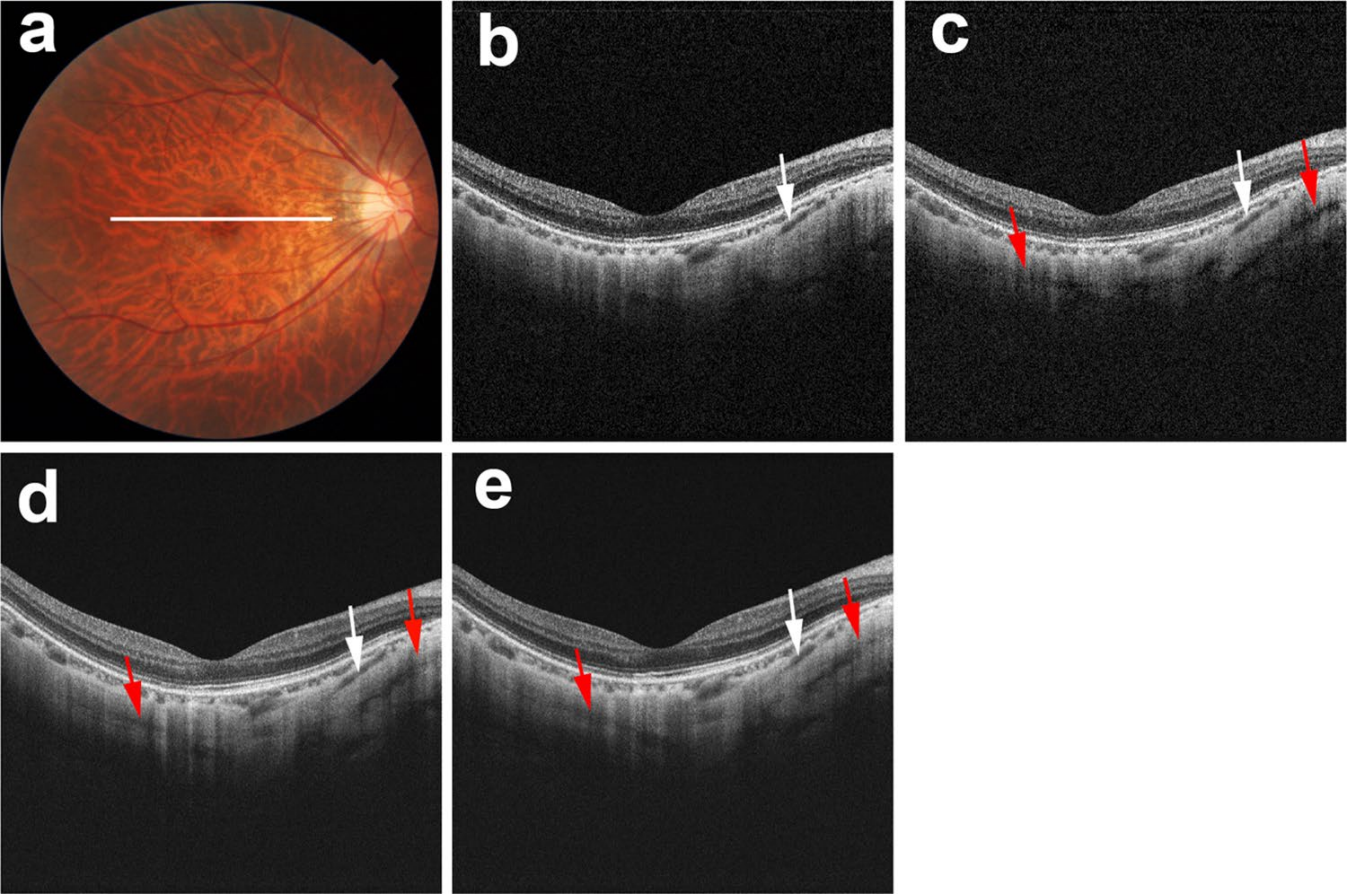
Scleral vessel artifacts in polarization-dependent OCT images and commercial OCT images in the same locations. The right eye of a 38-year-old woman with pathologic myopia is shown. White line in color fundus image (**a**) indicates the scan line for PD-OCT images and commercial OCT B-scan images (**b**–**e**). Polarization-independent OCT B-scan image (**b**) shows a real scleral vessel (white arrow). Polarization-dependent OCT B-scan image (**c**) and two repeated commercial OCT B-scan images (**d**, **e**) also show real scleral vessels (white arrows). Along with the real scleral vessel, polarization-dependent OCT B-scan image (**c**) shows scleral vessel artifacts (red arrows). Two repeated commercial OCT B-scan images (**d**, **e**) also show scleral vessel artifacts (red arrows) in locations similar to the locations in polarization-dependent OCT images.

**Figure 5:**
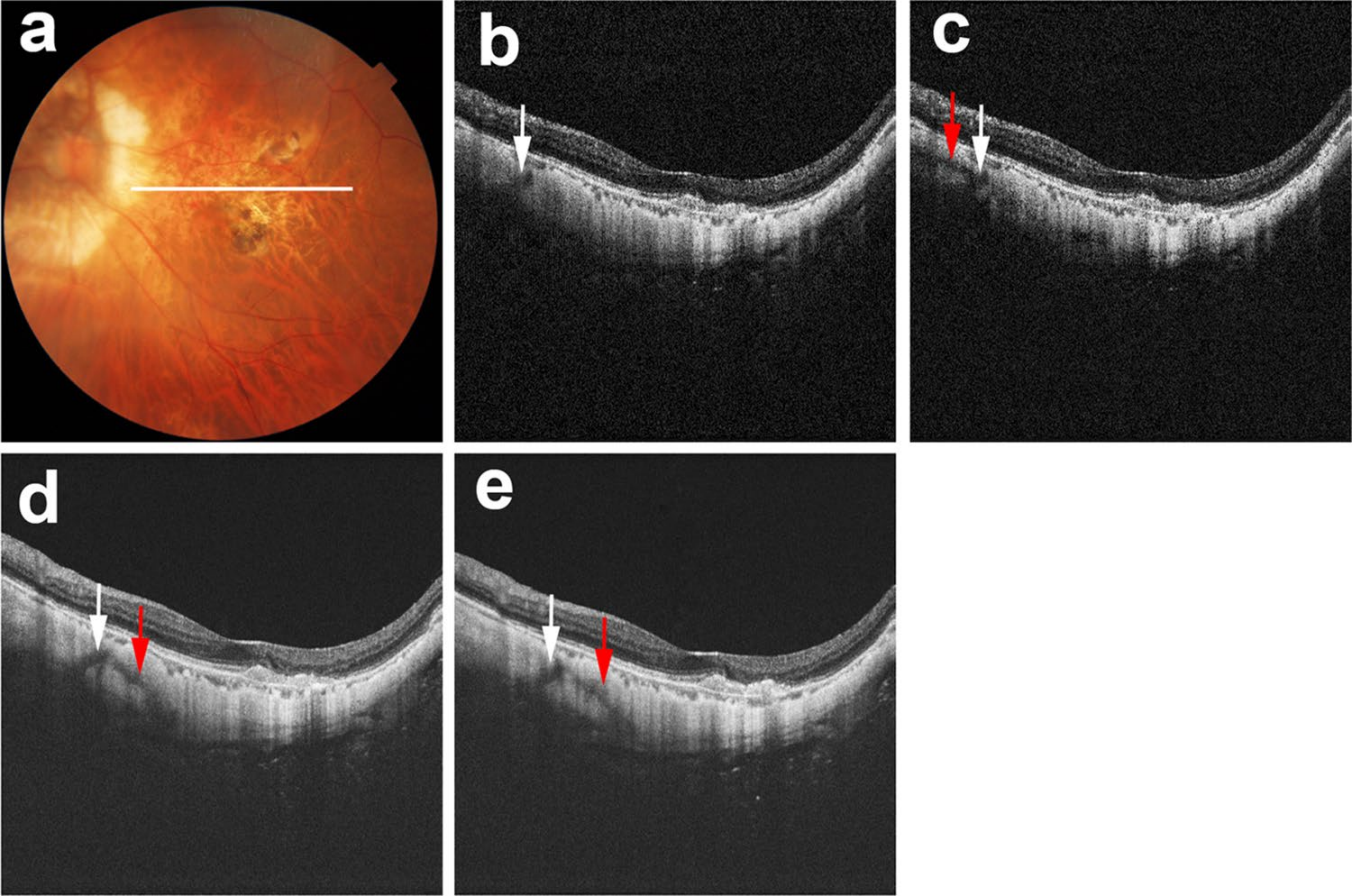
Scleral vessel artifacts in different locations in polarization-dependent OCT images and commercial OCT images. The left eye of a 75-year-old woman with myopic choroidal neovascularization in remission is shown. White line in color fundus image (**a**) indicates the scan line for PD-OCT images and commercial OCT B-scan images (**b**–**e**). Polarization-independent OCT B-scan image (**b**) shows a real scleral vessel (white arrow). Polarization-dependent OCT B-scan image (**c**) and two repeated commercial OCT B-scan images (**d**, **e**) also show real scleral vessels (white arrows). Along with the real scleral vessel, polarization-dependent OCT B-scan image (**c**) shows a scleral vessel artifact (red arrow). Two repeated commercial OCT B-scan images (**d**, **e**) show scleral vessel artifacts (red arrows) in locations that differ from the locations in polarization-dependent OCT images.

**Figure 6:**
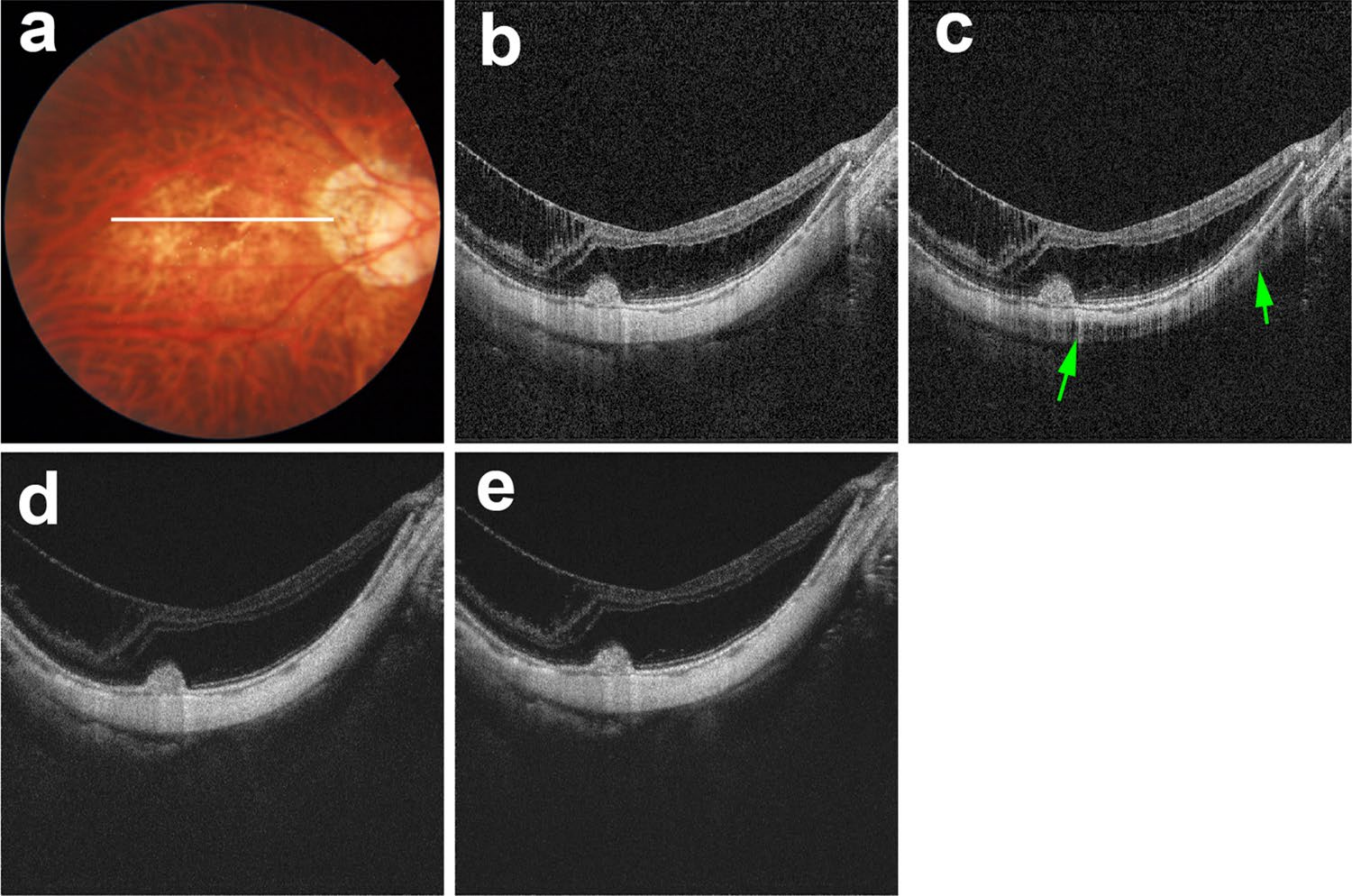
Low-intensity band artifact in a polarization-dependent OCT image. The right eye of a 33-year-old man who had macular retinoschisis with myopic choroidal neovascularization in remission is shown. White line in color fundus image (**a**) indicates the scan line for PD-OCT images and commercial OCT B-scan images (**b**–**e**). Polarization-dependent OCT B-scan image (**c**) shows a low-intensity band artifact (green arrows). No low-intensity band artifacts are present in a polarization-independent OCT B-scan image (**b**) or two repeated commercial OCT B-scan images (**d**, **e**).

Evaluation of five sets of macular B-scan images revealed that real linear low-intensity structures were present in polarization-independent OCT images; such structures corresponded with previously described scleral vessels (Fig. 2)^5,7,8,10,11^. Along with real scleral vessels, scleral vessel artifacts were present in polarization-dependent OCT images and commercial OCT images (Figs. 3, 4, 5). Figure 3 shows a representative case in which scleral vessel artifacts were only present in polarization-dependent OCT images. Figures 4 and 5 show representative cases in which scleral vessel artifacts were present in both polarization-dependent OCT images and commercial OCT images. All scleral vessel artifacts in commercial OCT images were present in two repeated measurements (Figs. 4 and 5). Table 1 shows the proportions of polarization-dependent OCT images and commercial OCT images that contained artifactual structures. The number of images that showed scleral vessel artifacts was significantly greater among polarization-dependent OCT images than among commercial OCT images (*P* < 0.001 for eyes and *P* < 0.001 for images, Pearson’s chi-squared test). Scleral vessel artifacts were observed in the same location in both commercial OCT images and polarization-dependent OCT images for 29 of 380 images (7.6%) (Fig. 4); they were observed in different locations in 34 of 380 images (8.9%) (Fig. 5). Along with scleral vessel artifacts, low-intensity band artifacts were present in polarization-dependent OCT images (Fig. 6, Table 1). In contrast, low-intensity band artifacts were not present in commercial OCT images. When evaluating the presence of artifacts in polarization-dependent OCT images and commercial OCT images, the kappa values for interobserver agreement were 0.97 (*P* < 0.001) and 0.95 (*P* < 0.001), respectively.

**Table 1:** Proportion of birefringence-derived artifacts in OCT B-scan images of the sclera.

	Polarization-dependent OCT	Commercial OCT
**Proportion of artifacts per eye (total 76 eyes)**		
Scleral vessel artifact	46 eyes (60.5%)	17 eyes (22.4%)
Low-intensity band artifact	22 eyes (28.9%)	0 eyes (0.0%)
**Proportion of artifacts per B-scan image (total 380 B-scan images)**		
Scleral vessel artifact	166 images (43.7%)	63 images (16.6%)
Low-intensity band artifact	63 images (16.6%)	0 images (0.0%)

OCT, optical coherence tomography.

In polarization-independent OCT, real scleral vessels were observed in 51 of 76 eyes (67.1%) and 149 of 380 B-scan images (39.2%). The mean number of real scleral vessels per image in polarization-independent OCT was 0.39 (standard deviation: 0.49, range: 0–1). The mean numbers of scleral vessels (including both real and artifactual structures) per image in polarization-dependent OCT and commercial OCT were 0.86 (standard deviation: 0.78, range: 0–3) and 0.56 (standard deviation: 0.62, range: 0–3), respectively. There were significantly fewer scleral vessels in polarization-independent OCT images than in polarization-dependent OCT images or commercial OCT images (*P* < 0.001 for polarization-dependent OCT and *P* = 0.007 for commercial OCT, Friedman and Bonferroni post hoc test). There were significantly fewer scleral vessels in commercial OCT images than in polarization-dependent OCT images (*P* < 0.001, Friedman and Bonferroni post hoc test). When evaluating the number of real scleral vessels in polarization-independent OCT images, as well as the total numbers of real and scleral vessel artifacts in polarization-dependent OCT images and commercial OCT images, the weighted kappa values for interobserver agreement were 0.84 (*P* < 0.001), 0.98 (*P* < 0.001), and 0.94 (*P* < 0.001), respectively.

**Figure 7:**
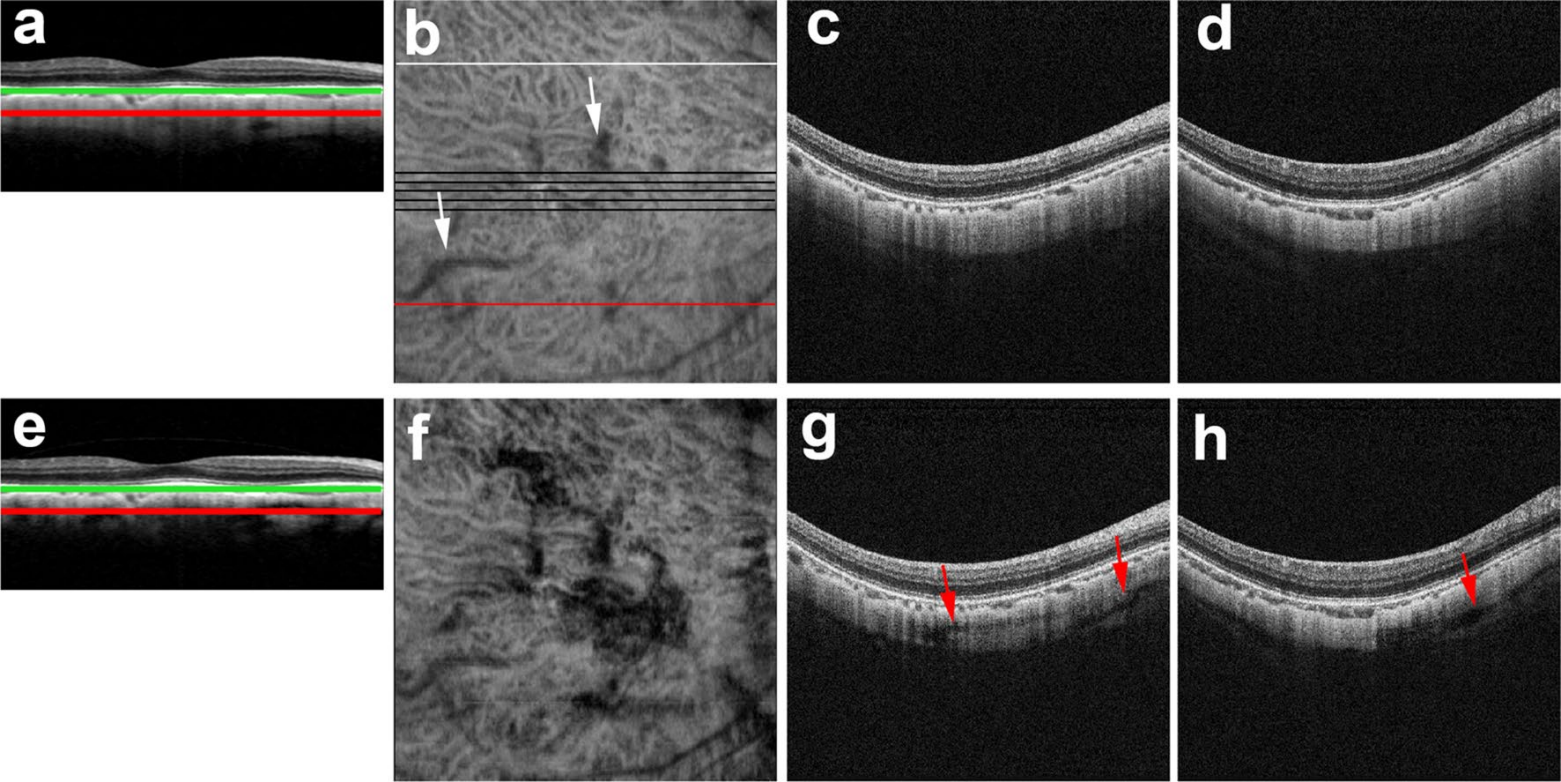
*En face* scleral PD-OCT images of the eye in Fig. 4. Polarization-independent OCT images (a–d) and polarization-dependent OCT images (**e**–**h**). PD-OCT B-scan images (**a**, **e**) were flattened with respect to Bruch’s membrane (green lines) to generate a reference plane (red lines) for *en face* scleral images (**b**, **f**). *En face* scleral image of polarization-independent OCT (**b**) shows real scleral vessels (white arrows) in the shadows of choroidal structures. Black lines indicate five sets of macular scan lines. White line indicates the upper scan line (**c**, **g**) and red line indicates lower scan line (**d**, **h**). *En face* scleral image of polarization-dependent OCT image (**f**) shows the distribution of artifactual low-intensity areas. By comparison with polarization-independent OCT B-scan images in upper (**c**) and lower (**d**) scan lines, scleral vessel artifacts in polarization-dependent OCT B-scan images are identified in both upper (**g**) and lower (**h**) scan lines (red arrows).

A comprehensive evaluation of the 256 PD-OCT B-scan images revealed that artifactual structures in polarization-dependent OCT B-scan images were present somewhere in the measurement area in all 76 eyes. Figure 7 shows *en face* scleral images of PD-OCT. The *en face* distribution of real scleral vessels could be detected in *en fac*e polarization-independent OCT images of areas within the shadows of choroidal structures^24^. The *en face* distribution of birefringence-derived artifacts could easily be identified as low-intensity areas in polarization-dependent OCT images. Artifactual structures were distributed throughout the measurement area.

## Discussion

There is increasing interest in analyses of pathologic myopia with respect to OCT images of scleral structure^5–12^. In this study, scleral vessel artifacts were identified in both polarization-dependent OCT images and commercial OCT images. Polarization-dependent OCT images showed scleral vessel artifacts in 46 of 76 eyes (60.5%), while commercial OCT images showed scleral vessel artifacts in 17 of 76 eyes (22.4%). The proportion of images that showed scleral vessel artifacts was significantly smaller among commercial OCT images than among polarization-dependent OCT images; however, the proportion of commercial OCT images that showed scleral vessel artifacts was not negligible. The presence of scleral vessels might be overestimated because of such artifacts. Additionally, low-intensity band artifacts were identified in polarization-dependent OCT images.

This study revealed considerable diversity in the patterns and locations of birefringence-derived artifacts. The directions of the scleral vessel artifacts also varied considerably. Furthermore, polarization-dependent OCT images showed low-intensity band artifacts. Structural birefringence in the sclera is associated with collagen fibril thickness, diameter, and orientation^14,15,25^. The orientation of collagen fibrils in the posterior human sclera is highly anisotropic and substantially varies throughout the tissue^1,26^; scleral collagen fibrils demonstrate considerable variation in terms of diameter and density^1,27^. Cross-sectional analysis has revealed that the myopic sclera demonstrates greater variation in terms of collagen fibril diameter, as well as an increased amount of unusual star-shaped fibrils^2^. These complex characteristics of scleral collagen fibrils in eyes with pathologic myopia might lead to a heterogeneous composition of birefringence-derived artifacts.

In this study, polarization-dependent OCT images showed birefringence-derived artifacts somewhere in the measurement area in all eyes. This finding highlights the inevitable presence of artifacts in polarization-dependent OCT images. The proportion of images with artifacts was significantly smaller among commercial OCT images than among polarization-dependent OCT images. Moreover, low-intensity band artifacts were observed in polarization-dependent OCT images but not in commercial OCT images. These findings suggested that birefringence-derived artifacts were partially resolved by using commercial OCT. To our knowledge, available retinal commercial OCT systems do not use polarization-diversity detection; they detect a polarization component that is identical to the polarization state of the reference beam^20^. Birefringence-derived artifacts can occur in both commercial OCT images and polarization-dependent OCT images. In commercial OCT images, the influence of birefringence-derived artifacts can be reduced by adjusting a polarization controller in the reference arm of the interferometer, thereby maximizing signal intensity^20^. In contrast, the PD-OCT hardware does not have this reference polarization optimization mechanism for several reasons^23^. First, for polarization diversity detection, the reference beam polarization should have 45-degree linear polarization with respect to the polarization optical components in the detector module. Therefore, we cannot align the reference polarization state in principle^23^. However, we can perform similar optimization by aligning the polarization state of the backscattered probe beam with a polarization controller. However, this PD-OCT device is not equipped with this mechanism because it is not necessary for its original purpose; i.e., to obtain the polarization-insensitive OCT image and polarization contrast (degree-of-polarization uniformity)^23^. In addition, it is not possible to mimic the automatic polarization alignment of the commercial DRI OCT Triton device because its details have not been disclosed.

Other factors that might influence the probability of birefringence-derived artifacts are the angle of incidence and the polarization state of the incident light. Scleral birefringence is affected by the relative angle between the beam and the sample; the extent of birefringence depends on the relative difference between the polarization state of the incident light and the optic axis orientation of the sclera^28,29^. These factors might lead to differences in the occurrence and pattern of artifacts between polarization-dependent OCT images and commercial OCT images. Indeed, the artifact patterns differed between commercial OCT and polarization-dependent OCT for nearly half of the images.

In polarization-independent OCT images, we identified real low-intensity structures that were previously described as scleral vessels. Along with real scleral vessels, polarization-dependent OCT images and commercial OCT images showed scleral vessel artifacts. Scleral vessels were previously investigated in association with variations in ciliary arteries^5,7,8,11^ or the presence of feeder vessels to sites of myopic choroidal neovascularization^10^. The presence of scleral vessel artifacts might lead to overestimation regarding the number of scleral vessels. These scleral vessel artifacts might be mistakenly regarded as ciliary arteries or feeder vessels in OCT images of the sclera; they might lead to erroneous interpretation of the processes that underlie pathologic myopia. The potential for scleral vessel artifacts should be considered in clinical evaluation with commercial OCT systems.

Polarization-dependent OCT images showed low-intensity band artifacts. In contrast, commercial OCT images did not show such artifacts. If low-intensity band artifacts are located at the posterior border of the sclera, they might influence measurements of scleral thickness. In the present study, commercial OCT successfully resolved such artifacts in images of most eyes, although the potential for low-intensity band artifacts should be considered when measuring scleral thickness.

The present study had some limitations. First, because of the small number of patients involved, this preliminary study was unable to fully evaluate some aspects of birefringence-derived scleral artifacts in OCT images. Further studies with larger numbers of patients are needed to comprehensively evaluate the clinical significance of such artifacts. Second, locations might have been inconsistent between PD-OCT B-scan images and commercial OCT B-scan images. Both polarization-independent OCT images and polarization-dependent OCT images were constructed from the same dataset; thus, these locations were closely matched. In contrast, commercial OCT images were obtained from independent measurements. This inconsistency might have affected the identification of artifacts in this study. Third, different scanning protocols (density and number of B-scans for averaging) were used for commercial OCT and PD-OCT. These differences affect the signal-to-noise ratio of OCT images and visibility of the sclera and therefore may have influenced our comparison between the commercial OCT and PD-OCT images. Fourth, the PD-OCT system in this study could not measure local birefringence at the sclera. Measurements by Jones matrix OCT and local birefringence are reportedly necessary for comprehensive evaluation of the influence of scleral birefringence^15^. Fifth, in previous clinical studies involving commercial OCT, full-thickness scleral structures were evaluated by averaging of multiple B-scan images. Because of this time-consuming procedure, previous studies omitted volumetric evaluation^5–10,12^. In the present study, we only evaluated five sets of macular B-scan images that were collected via commercial OCT in accordance with the built-in program of DRI-OCT Triton. In future studies, volumetric evaluation of commercial OCT images might provide more comprehensive data regarding artifactual structures. Sixth, this study used DRI-OCT Triton to represent commercial swept-source OCT systems; other swept-source OCT systems have become commercially available^11,12^. OCT images of the sclera have been obtained using spectral-domain OCT^7,8^. Because of differences in polarization state optimization, interferometer architecture, and image processing methods, birefringence-derived artifacts might differ among systems. Each commercial OCT system should be evaluated separately to determine how it is affected by birefringence-derived artifacts.

In conclusion, this study demonstrated that birefringence-derived scleral artifacts were present in commercial OCT images. These artifacts might cause overestimation of scleral vessels and erroneous interpretation of the pathogenesis of concurrent lesions in patients with pathologic myopia. In PD-OCT images, artifactual findings could easily be distinguished from real structures; hence, a PD-OCT system could improve the performance of scleral imaging in patients with pathologic myopia. PD-OCT may represent an effective tool to evaluate scleral structure in patients with pathologic myopia.

## Supplementary Information

Supplementary Information.

## Data Availability

The datasets used and/or analyzed during the current study available from the corresponding author on reasonable request.
