# Supplementary material for: Birefringence-derived scleral artifacts in optical coherence tomography images of eyes with pathologic myopia
Source: Sci Rep. 2022 Nov 16;12:19713. doi: 10.1038/s41598-022-23874-7 (PMC9669004; doi:10.1038/s41598-022-23874-7)
Supplement: Supplementary file 1 — Supplementary Information. [file 41598_2022_23874_MOESM1_ESM.pdf]

Birefringence-derived scleral artifacts in optical coherence tomography images of eyes with pathologic myopia

Masahiro Miura, Shuichi Makita, Yoshiaki Yasuno, Atsuya Miki, Rei Nemoto, Hiroyuki Shimizu, Shinnosuke Azuma, Toshihiro Mino, Tatsuo Yamaguchi

Supplementary Table S1.

Summary of patient characteristics and artifactual structures in polarization-dependent OCT images and commercial OCT images.

Patient	Eye	Age (years)	Sex	Axial length (mm)	Artifactual structures in polarization-dependent OCT images	Artifactual structures in commercial OCT images
1	R	66	F	26.61	Scleral vessel artifacts	None
1	L	66	F	27.35	Scleral vessel artifacts, low-intensity band artifact	None
2	R	37	M	30.02	None	None
3	R	74	M	27.47	Scleral vessel artifacts, low-intensity band artifact	None
3	L	74	M	28.14	Scleral vessel artifacts, low-intensity band artifact	None
4	R	36	F	27.34	Scleral vessel artifacts	Scleral vessel artifacts
4	L	36	F	27.18	Low-intensity band artifact	None
5	R	81	M	29.68	Low-intensity band artifact	None
5	L	81	M	29.87	Scleral vessel artifacts	None
6	R	70	F	27.46	None	None
6	L	70	F	26.52	Scleral vessel artifacts	None
7	R	64	F	30.33	Scleral vessel artifacts	Scleral vessel artifacts
7	L	64	F	30.73	Scleral vessel artifacts	None
8	R	75	F	31.18	Scleral vessel artifacts	None
8	L	75	F	30.43	Scleral vessel artifacts	Scleral vessel artifacts
9	R	68	M	26.75	Scleral vessel artifacts	Scleral vessel artifacts
9	L	68	M	28.61	None	None
10	R	59	F	29.43	None	None
10	L	59	F	27.49	Scleral vessel artifacts	None
11	R	23	F	28.25	Scleral vessel artifacts	None
11	L	23	F	28.68	Scleral vessel artifacts	None
12	R	66	F	29.23	Scleral vessel artifacts	None
13	R	67	F	29.71	Scleral vessel artifacts, low-intensity band artifact	None
13	L	67	F	29.85	Scleral vessel artifacts, low-intensity band artifact	None
14	R	72	F	26.81	None	None
14	L	72	F	26.93	None	None
15	R	57	F	31.84	None	None
15	L	57	F	31.97	None	None

16	R	69	F	31.22	None	None
16	L	69	F	29.28	None	None
17	R	57	F	30.82	Scleral vessel artifacts	Scleral vessel artifacts
17	L	57	F	29.93	Scleral vessel artifacts	None
18	R	81	F	28.52	None	None
18	L	81	F	28.51	Scleral vessel artifacts	None
19	R	63	M	27.09	Scleral vessel artifacts	None
19	L	63	M	26.79	None	None
20	R	51	F	28.89	None	None
20	L	51	F	28.68	Scleral vessel artifacts	None
21	R	66	F	28.02	None	None
22	R	78	F	28.99	None	None
23	R	57	F	29.21	Scleral vessel artifacts	None
23	L	57	F	29.22	Scleral vessel artifacts	None
24	R	72	F	29.35	Scleral vessel artifacts	None
25	R	71	F	31.24	Scleral vessel artifacts	None
25	L	71	F	31.56	Scleral vessel artifacts	Scleral vessel artifacts
26	R	82	F	31.77	Low-intensity band artifact	Scleral vessel artifacts
27	R	56	F	29.27	None	None
27	L	56	F	29.04	Scleral vessel artifacts	Scleral vessel artifacts
28	R	76	F	26.54	Scleral vessel artifacts, low-intensity band artifact	None
28	L	76	F	26.73	Scleral vessel artifacts, low-intensity band artifact	Scleral vessel artifacts
29	R	80	F	28.82	Low-intensity band artifact	None
29	L	80	F	29.27	Low-intensity band artifact	None
30	R	80	F	31.91	Scleral vessel artifacts	None
30	L	80	F	31.79	Low-intensity band artifact	None
31	R	38	F	29.32	Scleral vessel artifacts, low-intensity band artifact	None
31	L	38	F	28.08	Scleral vessel artifacts	None
32	R	66	M	26.89	Scleral vessel artifacts	Scleral vessel artifacts
32	L	66	M	26.68	Low-intensity band artifact	None
33	R	51	M	32.07	None	None
33	L	51	M	31.83	None	None
34	R	53	F	27.16	Scleral vessel artifacts	None
34	L	53	F	28.56	Scleral vessel artifacts	Scleral vessel artifacts
35	R	70	M	29.79	Scleral vessel artifacts, low-intensity band artifact	Scleral vessel artifacts
35	L	70	M	29.91	Scleral vessel artifacts	None
36	L	73	F	30.57	Low-intensity band artifact	None
37	R	65	F	27.16	Low-intensity band artifact	None
37	L	65	F	28.03	Low-intensity band artifact	None
38	R	59	F	26.59	Scleral vessel artifacts, low-intensity band artifact	None

38	L	59	F	26.68	Scleral vessel artifacts	Scleral vessel artifacts
39	R	33	M	32.38	Low-intensity band artifact	None
39	L	33	M	31.84	Low-intensity band artifact	None
40	R	50	F	29.98	Scleral vessel artifacts	Scleral vessel artifacts
40	L	50	F	29.04	Scleral vessel artifacts	Scleral vessel artifacts
41	L	56	M	28.40	Scleral vessel artifacts	Scleral vessel artifacts
42	R	38	F	27.17	Scleral vessel artifacts	Scleral vessel artifacts
42	L	38	F	26.88	Scleral vessel artifacts	None
